# The STING1 network regulates autophagy and cell death

**DOI:** 10.1038/s41392-021-00613-4

**Published:** 2021-06-02

**Authors:** Ruoxi Zhang, Rui Kang, Daolin Tang

**Affiliations:** Department of Surgery, UT Southwestern Medical Center, Dallas, TX USA

**Keywords:** Cell biology, Immunology

## Abstract

Cell death and immune response are at the core of life. In past decades, the endoplasmic reticulum (ER) protein STING1 (also known as STING or TMEM173) was found to play a fundamental role in the production of type I interferons (IFNs) and pro-inflammatory cytokines in response to DNA derived from invading microbial pathogens or damaged hosts by activating multiple transcription factors. In addition to this well-known function in infection, inflammation, and immunity, emerging evidence suggests that the STING1-dependent signaling network is implicated in health and disease by regulating autophagic degradation or various cell death modalities (e.g., apoptosis, necroptosis, pyroptosis, ferroptosis, mitotic cell death, and immunogenic cell death [ICD]). Here, we outline the latest advances in our understanding of the regulating mechanisms and signaling pathways of STING1 in autophagy and cell death, which may shed light on new targets for therapeutic interventions.

## Introduction

Pathogen-associated molecular patterns (PAMPs) derived from microorganisms and damage-associated molecular patterns (DAMPs) produced by host cells are recognized by pattern recognition receptors (PRRs), which play a fundamental role in innate immunity during infection and tissue damage^1,2^. Major PAMPs include microbial nucleic acids (DNA and RNA) and membrane components (e.g., lipopolysaccharide [LPS]), whereas the host DNA is an important DAMPs. Dysregulation of DNA-sensing pathways is implicated in various diseases, such as autoimmune diseases and cancer. In 2008 and 2009, stimulator of interferon response cGAMP interactor 1 (STING1, also known as STING, TMEM173, MITA, or MPYS) was identified by multiple groups as a key adapter in DNA-mediated innate immunity^3–6^. In 2013, Dr. Chen’s group ultimately identified that cyclic GMP-AMP synthase (CGAS) is a direct cytosolic DNA sensor that elicits robust innate immune responses through STING1^7^. These studies have established a new DNA recognition pathway in the innate immune system^8,9^.

**Fig. 1:**
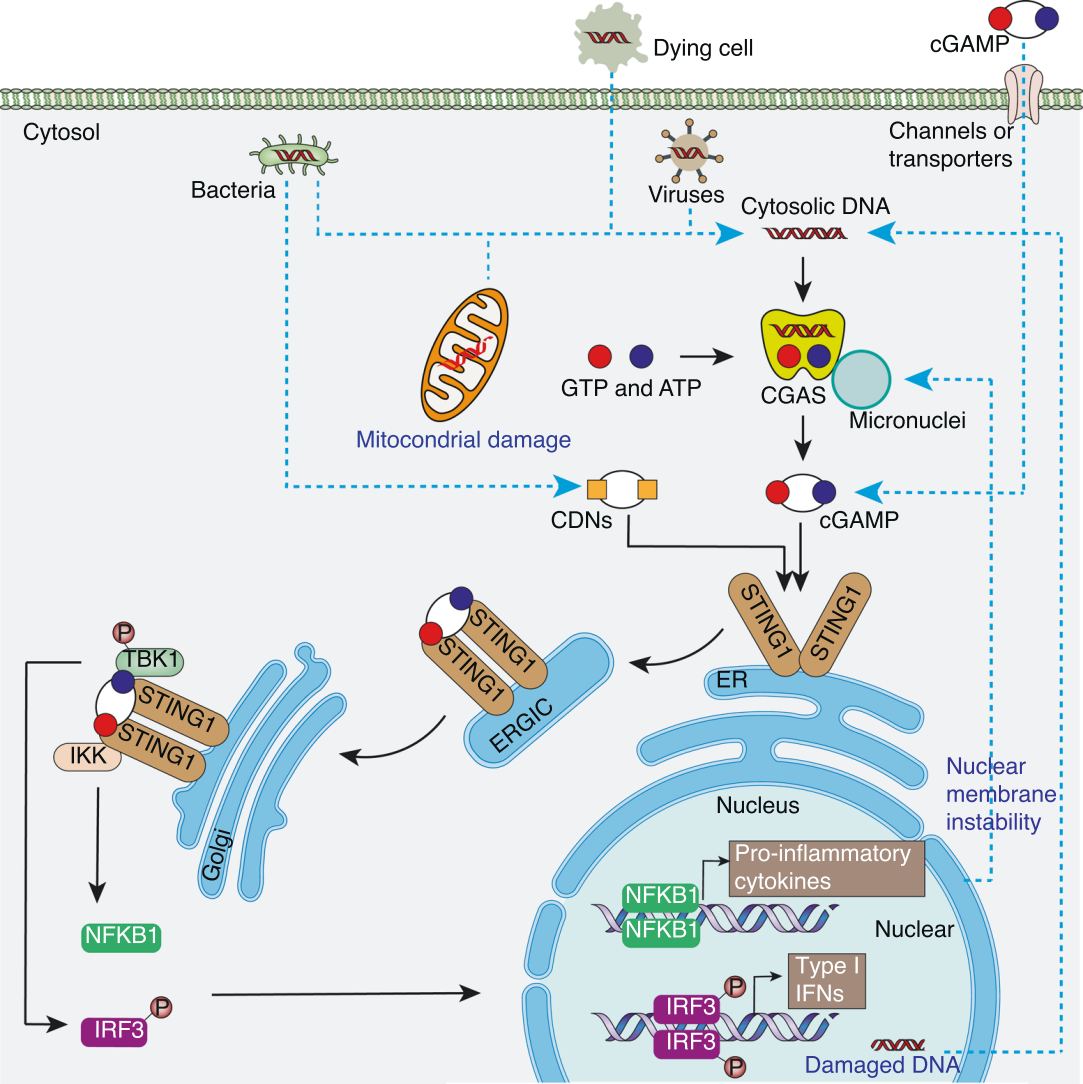
The CGAS-STING1 pathway. A critical cytosolic DNA sensor, CGAS elicits robust innate immune responses through the production of 2′3′-cGAMP, which binds and activates STING1 in response to DNA from pathogens or hosts. In addition, bacteria-produced CDNs can directly activate STING1 in a CGAS-independent manner. Activated STING1 exits the ER membrane and travels to Golgi via the ERGIC, leading to the production of type I IFNs by activating the TBK1-IRF3 pathway. STING1 also activates NFKB1-dependent pro-inflammatory cytokine expression. CDNs cytosolic cyclic dinucleotides, cGAMP cyclic GMP-AMP, CGAS cyclic GMP-AMP synthase, ER endoplasmic reticulum, ERGIC endoplasmic reticulum-Golgi intermediate compartment, IFN interferon, IKK IκB kinase, IRF3 interferon regulatory factor 3, NFKB1 nuclear factor kappa B subunit 1, STING1 stimulator of interferon response cGAMP interactor 1, TBK1 TANK binding kinase 1

STING1 is an evolutionarily conserved transmembrane protein that localizes to the endoplasmic reticulum (ER) membrane in immune and non-immune cells. As an adapter protein, STING1 can be directly activated by bacterial cyclic dinucleotides (CDNs, such as cyclic-di-GMP and cyclic-di-AMP) or the second messenger cyclic guanosine monophosphate-adenosine monophosphate (cGAMP)^10,11^, a process that is involved in inflammation and immune response by producing type I interferons (IFNs) and pro-inflammatory cytokines^3,8^. The cGAMP is produced from CGAS, which detects and binds DNA substrates from invading pathogens (e.g., DNA viruses, retroviruses, and bacteria) or damaged hosts (including mitochondrial DNA [mtDNA] and nuclear DNA [nDNA]) during various stresses^12^ (Fig. 1). In addition, cGAMP or host DNA can be transferred between cells and activate STING1 through channels and transporters^13–15^, dying cell debris, or DNA-containing extracellular vesicles^16^. In addition to cytoplasmic CGAS, plasma membrane receptors (such as epidermal growth factor receptor [EGFR] and ALK receptor tyrosine kinase [ALK]) can also activate STING1 in response to exogenous cGAMP or CDNs in immune cells^17^. Overall, these findings indicate that the activation of STING1 can be induced by both CGAS-dependent and -independent pathways.

After activation, STING1 on the ER undergoes oligomerization^18^, leaving the ER through the chromosome X open reading frame 56 (CxORF56) and coat protein complex II (COPII)^19–21^, and finally translocating to the Golgi apparatus through the endoplasmic reticulum-Golgi intermediate compartment (ERGIC)^22^ (Fig. 1). In the Golgi, palmitoylated STING1 recruits TANK binding kinase 1 (TBK1)^23,24^, and TBK1 further transphosphorylates the C-terminal tail of STING1 to recruit interferon regulatory factor 3 (IRF3) for phosphorylation^25^. Phosphorylated IRF3 translocates to the nucleus and triggers the expression of immune stimulated genes (ISGs) and type I IFNs, resulting in the activation and migration of immune cells (including dendritic cells [DCs], T cells, and natural killer [NK] cells) to the target cells^26^. Alternatively, STING1 also activates nuclear factor kappa B subunit 1 (NFKB1)-driven inflammatory cytokine (e.g., tumor necrosis factor [TNF] and interleukin 6 [IL6]) production. As a negative feedback mechanism, the degradation of cGAMP, CGAS, or STING1 at various levels can limit type I IFN responses^27,28^. Functionally, the activation of this classical STING1 pathway bridges innate and adaptive immunity in response to PAMPs or DAMPs. Consequently, an insufficient or excessive activation of the STING1 pathway is implicated in various pathological conditions, such as tumorigenesis, infection, disseminated intravascular coagulation, autoimmune conditions, and tissue damage^3,5,6,9,17,29–34^.

Beyond the canonical role of STING1 in mediating cytokine production, growing evidence highlights the emerging role of STING1 in regulating autophagy and cell death. In this review, we focus on new discoveries about the regulation mechanisms and outcomes of STING1 in autophagy and cell death, which provide another framework to understand the biological function of STING1 in health and disease.

## STING1 in autophagy

Autophagy is a degradation process that can be classified into three types: macroautophagy, microautophagy, and chaperone-mediated autophagy^35^. Macroautophagy (hereafter referred to as autophagy) is the most studied form of autophagy and regulates homeostasis by either promoting survival or inducing cell death^36–38^. Autophagy is also closely related to inflammation and immune response^39^. Here, we not only outline the basic process of autophagy, but also discuss the interaction between STING1 activation and autophagy induction.

### Membrane dynamics during autophagy

The process of autophagy involves the formation of membrane structures, especially phagophores, autophagosomes, and autolysosomes^38^. This dynamic membrane process can be roughly divided into five consecutive phases: initiation, nucleation, expansion, fusion, degradation, and recycling, and is controlled by autophagy-related (ATG) family proteins, kinases, and lipid metabolism^36,37,40,41^. The initiation step is triggered by inhibiting mechanistic target of rapamycin kinase (MTOR) or activating AMP-activated protein kinase (AMPK), leading to the assembly and activation of the ULK complex (including unc-51-like autophagy activating kinase 1/2 [ULK1/2, orthologs of yeast Atg1], ATG13, ATG101, and RB1 inducible coiled-coil 1 [RB1CC1/FIP200]) and subsequent the class III phosphatidylinositol 3-kinase (PtdIns3K) complex (containing PIK3C3 [phosphatidylinositol 3-kinase catalytic subunit type 3, an ortholog of yeast Vps34], beclin 1 [BECN1, a mammalian homolog of yeast Vps30/Atg6], ATG14L [autophagy-related protein 14-like protein], and PIK3R4 [phosphoinositide 3-kinase regulatory subunit 4, a mammalian homolog of yeast Vps15]). The PtdIns3K complex-mediated production of phosphatidylinositol 3-phosphate (PI3P) leads to the recruitment of PI3P-binding ATG proteins, WD repeat domain phosphoinositide-interacting (WIPI) proteins, and ATG9-containing vesicles, thereby supporting phagophore nucleation (mostly at the ER). In addition to regulating autophagy, BECN1 is also a multifunctional protein in cell death through different binding partners^42,43^. To promote the expansion and closure of the phagophore membrane, two ubiquitin-like conjugation pathways are needed to mediate the conjugation of microtubule-associated protein 1 light chain 3 (MAP1LC3, an ortholog of yeast Atg8) and phosphatidylethanolamine (PE). This process is called MAP1LC3 lipidation, which is controlled by many ATGs. The E1-like enzyme ATG7, the E2-like enzyme ATG3, and the cysteine protease ATG4 lead to the formation of MAP1LC3-II. The ATG7 and the E2-like enzyme ATG10 catalyze the formation of the ATG12-ATG5-ATG16L1 complex, which binds to WIPI and functions as an E3-like ligase to mediate the lipidation of MAP1LC3-II. Lipidated MAP1LC3-II also enables the assembly of phagophores with autophagic cargo receptors (e.g., sequestosome 1 [SQSTM1/p62]) to engulf cytoplasmic materials. The closure of the phagophore results in the formation of double-membrane structures, namely autophagosomes. Autophagosomes finally fuse with lysosomes to produce autolysosomes, where the cargoes are degraded^44^. The subsequent degradation of autophagic cargoes by lysosomal hydrolases leads to the release of nutrients and materials back to the cytoplasm for reuse. Overall, from yeasts to plants and animals, membrane dynamics during autophagy are highly conserved.

### STING1-mediated autophagy prevents pathogen infection

Since it was first documented that ATG9a and STING1 co-localized in vesicles and restricted dsDNA-induced IFN production in mouse embryonic fibroblasts (MEFs)^45^, accumulating evidence has enabled us to understand how STING1 plays context-dependent roles in the induction and regulation of autophagy under different stresses. In general, pathogenic DNA can activate STING1 and subsequent autophagy to restrict pathogen infection by removing bacteria and viruses^46–48^, as described below.

During *Mycobacterium tuberculosis* infection, STING1 is activated by CGAS, triggering ubiquitin-mediated selective autophagy (namely xenophagy) by SQSTM1, ATG5, and TBK1 in macrophages to eliminate *M. tuberculosis*^11,47–50^. In contrast, c-di-AMP induces STING1-dependent autophagy via MTOR inactivation in macrophages during Gram-positive bacterial infection^51^, whereas the deletion of ULK1, RB1CC1, and ATG14L can limit MAP1LC3 lipidation during this process. Similar to bacterial infection, during herpes simplex virus 1 (HSV-1) infection, cytosolic viral DNA triggers STING1-dependent autophagy in bone marrow-derived DCs^46^. Further studies using *Sting1* mutant mice suggest that STING1-mediated autophagy (but not STING1-dependent IFN production) is responsible for anti–HSV-1 responses^52^, although HSV may also counter this STING1-mediated autophagy induction in vivo^53^. This autophagy-dependent antiviral function is also observed in human rhinovirus (HRV)-infected HeLa cells after treatment with the STING1 agonist dimeric amido-benzimidazole (diABZI)^54^, although it is unclear whether CGAS is required for this process.

STING1-mediated autophagy during infection is also related to NF-κB signaling. For example, NF-κB-mediated STING1 expression triggers autophagy in the adult fly brain against Zika virus (ZIKV) infection^55^. Moreover, NF-κB mediates the expression of DNA damage-regulated autophagy modulator (DRAM1), thereby inducing selective autophagy in zebrafish and human macrophages during *M. tuberculosis* infection, and this process is positively regulated by STING1^56^. STING1 can also recognize and degrade the contents of lytic *Chlamydiae trachomatis* in HeLa cells through a selective autophagy pathway^57^. Moreover, the antibacterial protein IL-26 secreted by Th17 cells can induce STING1-dependent autophagy to eliminate invasive *Mycobacterium leprae* in THP-1 cells^58^.

Together, STING1-dependent autophagic clearance of invading pathogens may be an important host defense mechanism against infection. However, the interplay between STING1-dependent xenophagy and cytokine production remains uncertain.

### STING1-mediated autophagy limits tumor growth and transformation

In addition to promoting survival, excessive autophagy may also lead to cell death, known as autophagy-dependent cell death (ADCD)^59^. Although ADCD is also a type of regulated cell death (RCD) according to the recommendation of the Nomenclature Committee on Cell Death^60^, it relies on components of autophagic machinery and takes place in the context of apoptosis^61^, necroptosis^62–64^, and ferroptosis^65–69^. Two studies on pancreatic cancer cells have linked DNA damage, STING1 activation, and autophagy-dependent ferroptosis. The nucleoside analog zalcitabine (an antiviral drug) induces oxidative mtDNA damage and the release of mtDNA into cytosol, resulting in the activation of the CGAS-STING1 pathway, which in turn induces autophagy-dependent ferroptosis and suppresses pancreatic tumor growth in mice^70^. In addition to mtDNA, the release of nDNA to cytosol caused by nuclear cathepsin B (CTSB)-mediated genomic DNA damage activates STING1-dependent autophagy and subsequent glutathione peroxidase 4 (GPX4) degradation, leading to ferroptotic cell death in human pancreatic cancer cells^71^. These findings provide an example of how lysosomal proteins can drive STING1 activation for autophagy-dependent ferroptosis.

Replicative crisis is a senescence-independent process, characterized by chromosomal instability^72^. It functions as a potent tumor suppressor against oncogenic transformation and tumorigenesis and culminates in extensive cell death, which is modulated by telomeric damage signals^72,73^. When replicative crisis occurs, increased telomere DNA damage also initiates CGAS-STING1-dependent ADCD to limit genome instability in human mammary epithelial cells and IMR90^E6E7^ cells^74^. These results demonstrate tumor suppressive roles for STING1-dependent ADCD during transformation. Accordingly, *Burkholderia pseudomallei*-induced cell fusion triggers mitotic events and subsequent micronuclei formation, which leads to CGAS-STING1 activation and ultimately STING1-dependent ADCD in macrophages^75^, suggesting that ADCD acts as a natural defense against cellular transformation and unnatural cellular fusions.

Overall, these in vitro and in vivo data indicate that STING1 activation induced by host DNA damage can trigger ADCD to remove cancer cells or other stressed cells. It will be interesting to see whether the activation of STING1-dependent autophagy plays a direct role in degrading DNA or micronucleus in damaged cells, a process called DNAphagy.

### The mechanism of STING1 regulates the autophagy pathway

**Fig. 2:**
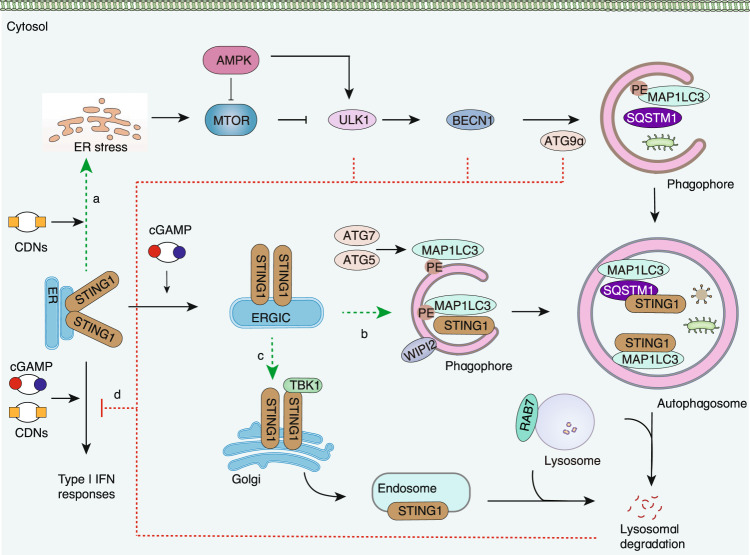
Crosstalk between STING1 and autophagy. **a** The activation of STING1 by bacterial CDNs triggers autophagy through ER stress or the MTOR-ULK1-BECN1 pathway. **b** cGAMP binds to STING1, causing it to be translocated from the ER to the ERGIC and Golgi. The ERGIC serves as a membrane source for the recruitment and lipidation of MAP1LC3 through an ATG5-, ATG7-, and WIPI2-dependent mechanism, leading to autophagosome formation. Autophagosomes engulfing DNA and pathogens target STING1 in an SQSTM1-dependent or -independent manner. **c** Activated STING1 can translocate to endosomes through the trans-Golgi network. Both the endosomes and autophagosomes fused with lysosomes require RAB7A GTPase. **d** STING1-mediated IFN response is also negatively regulated by autophagic degradation or ATGs (including ULK1, BECN1, and ATG9a). AMPK AMP-activated protein kinase, ATG autophagy-related, BECN1 beclin 1, CDNs cytosolic cyclic dinucleotides, cGAMP cyclic GMP-AMP, ER endoplasmic reticulum, ERGIC endoplasmic reticulum-Golgi intermediate compartment, IFN interferon, MAP1LC3 microtubule-associated protein 1 light chain 3, MTOR mechanistic target of rapamycin kinase, PE phosphatidylethanolamine, PIK3C3 phosphatidylinositol 3-kinase catalytic subunit type 3, RAB7A, member RAS oncogene family, SQSTM1 sequestosome 1, STING1 stimulator of interferon response cGAMP interactor 1, TBK1 TANK binding kinase 1, ULK1 unc-51–like autophagy activating kinase 1, WIPI2 WD repeat domain phosphoinositide-interacting 2

The autophagy mediated by STING1 is a selective process that requires specific signals and regulators in a context-dependent manner (Fig. 2). After binding to cGAMP, STING1 leaves the ER and is transported to the ERGIC, which serves as a membrane source for autophagosome biogenesis^21^. STING1-mediated autophagosome formation is generally independent of the activation of TBK1 and IRF3^21,22,52,76^ as well as ULK1, BECN1, and PIK3C3^21,76^. Moreover, although ATG9a is a regulator of STING1 trafficking^45^ and nucleation of autophagy^77^, it is dispensable for STING1-dependent autophagy^76^. These findings suggest that STING1 initiates a noncanonical form of autophagy driving autophagosome formation. Subsequent studies have highlighted that WD repeat domain, phosphoinositide-interacting 2 (WIPI2), ATG5, and ATG7 are required for STING1-induced autophagosome formation^21^, whereas RAB7A promotes the transport of STING1 to lysosomes through autophagosomes and endosomes^21,27^. Finally, STING1 can be self-degraded by autophagy machinery, which requires the use of the TBK1 signal and autophagy receptor SQSTM1 in MEFs and THP-1 cells^78^, while SQSTM1 and TBK1 are required for STING1- and ubiquitin-mediated selective autophagy targeting *M. tuberculosis* in macrophages^48^. Alternatively, since TBK1 is not required for STING1-induced autophagy in HeLa cells, STING1 can function as an autophagy receptor itself that directly binds with MAP1LC3 via its LIR motifs in a SQSTM1-independent manner^76^. Consistently, the lack of ATG5 or WIPI2 abolishes SQSTM1 degradation (but not STING1 degradation) in cGAMP-treated BJ cells^21^. In addition, extracellular SQSTM1 is an inflammatory mediator in mice with bacterial infections, and the lysosome-dependent release of SQSTM1 induced by LPS in macrophages is initiated by STING1-TBK1–mediated phosphorylation of SQSTM1 on Ser 403^31^. These findings confirm that STING1 is not only an autophagy substrate, but also a modulator of autophagy during infection, which shapes host defense response, coupled to signals via SQSTM1 release.

In other cases, a BECN1-related PtdIns3K complex may contribute to STING1-dependent ADCD in HeLa cells during *Chlamydiae* infection^57^. Bacterial c-di-AMP activates STING1 and triggers ER stress, leading to MTOR inactivation and subsequent ER-phagy in macrophages^51^. Considering that STING1 is widely involved in ER calcium homeostasis^79^ and the unfolded protein response^80^, it is possible that STING1 activation may also trigger autophagy through ER stress and the MTOR-BECN1 pathway (Fig. 2).

Moreover, the activation of STING1 by cGAMP also induces V-ATPase-dependent MAP1LC3 lipidation to single-membrane perinuclear vesicles through ATG16L1^81^, suggesting an additional function of STING1 in membrane dynamics. In line with this, STING1 was observed to increase PI3P production and ER membrane curvature and to cluster at ER curvature-rich regions after cGAMP stimulation^19^. HRV replication also requires that STING1 is expressed on phosphatidylinositol 4-phosphate (PI4P)-enriched replication organelles^82^. Given that the IFN-dependent C-terminal tail region is not required for STING1 to induce autophagy^21,52^, it is possible that STING1 contributes to a wide range of cellular functions via membrane dynamics and ER exit trafficking in an IFN-independent manner. Of note, the activation of STING1 by lipotoxicity induced by saturated fatty acids may inhibit hepatocyte autophagy, which is related to the increase of SQSTM1 phosphorylation and oxidative stress^83^. The precise role of STING1 in autophagy regulation should be carefully evaluated under different stress conditions.

### The mechanism of autophagy modulating the STING1 pathway

The function of STING1 in immunity is also dually regulated by autophagic degradation or components of autophagy machinery. On one hand, autophagy restricts the activation of the STING1 pathway through multiple mechanisms (Fig. 2). First, autophagy can remove radiation-caused cytosolic mtDNA accumulation in breast cancer cells^84^. Second, AMPK and ULK1 mediate the phosphorylation of STING1 on S366, leading to the degradation of STING1 and inhibition of IFN production^85,86^. Consistently, the inactivation of ULK1 enhances the STING1-mediated innate immune response in keratinocytes under UV treatment^87^. Third, the autophagic degradation of STING1 in lysosomes by V-ATPase diminishes STING1 activation^21,27^. In contrast, blocking STING1 degradation by the V-ATPase inhibitor bafilomycin A enhances STING1-mediated immune signaling and antitumor response. Fourth, BECN1 is a negative regulator of STING1, which partially affects the phosphorylation of STING1 by binding to STING1^88,89^. Given that BECN1 and STING1 can bind to ER Ca^2+^ channel inositol 1,4,5-trisphosphate receptors (ITPRs/InsP3Rs) to promote the release of Ca^2+^ from the ER^29,79,90^, they may form a complex on the ER that controls autophagosome formation by ITPR-mediated Ca^2+^ signaling. Fifth, the pharmacological or genetic inhibition of ATG9a augments STING1-dependent IFN production^45,91^. On the other hand, a few studies show that the knockdown of PIK3C3 inhibits STING1 trafficking and activation^85^. Thus, it remains a challenge to distinguish the autophagy-dependent and -independent roles of ATG in regulating the activation of the STING1 pathway.

## STING1 in cell death

RCD is an active process involving tightly structured signal transduction cascades, molecularly defined effector mechanisms, and membrane repair machineries^92–97^. It has many forms, such as but not limited to apoptosis, necroptosis, pyroptosis, ferroptosis, ADCD, and immunogenic cell death (ICD)^98^, and plays a vital role in various physiological or pathological processes, including tissue regeneration, infection, immunity, and tumorigenesis^99^. Such a homeostatic function not only reflects the elimination of damaged or aged cells, but also the ability of dying cells to expose or release DAMPs that activate immune responses^100–103^. Recent evidence indicates that STING1 not only mediates cell death, but also plays a role in recognizing and amplifying the immune response induced by dying cells.

### STING1 in apoptosis

Apoptosis is usually a type of immune-silent RCD because it has a limited ability to release contents and can be quickly cleared by phagocytes (a process also known as efferocytosis). The activation of apoptosis generally includes two distinct pathways: the death receptor (or extrinsic) and the mitochondrial (or intrinsic) pathways. In many cases, caspases (cysteine-dependent proteases), such as caspase 3 (CASP3), CASP6, and CASP7, are effectors of apoptosis by mediating the cleavage of structural proteins^104,105^. The mitochondrial apoptosis pathway is initiated by various stresses, leading to increased mitochondrial outer membrane permeability (MOMP) and the release of mitochondrial proteins, such as cytochrome C, somatic (CYCS). Cytosolic CYCS binds to apoptotic peptidase activating factor 1 (APAF1), leading to the activation of CASP9 and subsequent executioner caspases CASP3 and CASP7, and ultimately causing apoptosis^60,106^. The interaction of anti-apoptotic or pro-apoptotic BCL2 family members orchestrates MOMP and has become a therapeutic target in clinical trials^107^. The death receptors include Fas receptors, TNF receptors, and TNF-related apoptosis-inducing ligand (TRAIL) receptors, which can recruit the adapter protein Fas associated via death domain (FADD) to active CASP8 or CASP10, and ultimately lead to apoptosis by activating CASP3 or CASP7^108^. In contrast, the mechanism and function of caspase-independent apoptosis remains poorly understood.

**Fig. 3:**
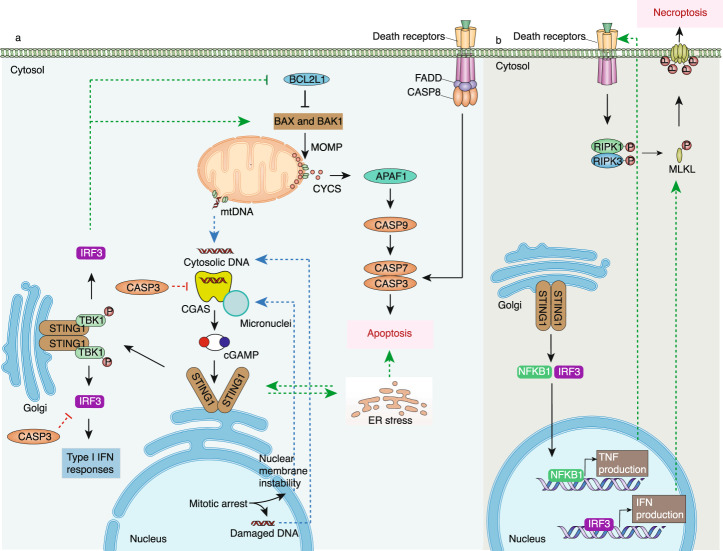
STING1 in apoptosis, mitotic cell death, and necroptosis. **a** In response to mtDNA, nDNA, or ER stress, STING1 activates IRF3. IRF3 can increase BAX/BAK1-mediated MOMP by forming an IRF3-BAX complex or inhibiting BCL2L1, leading to caspase activation and ultimately apoptosis. In turn, increased apoptosis double-regulates the STING1-IRF3 pathway by releasing mtDNA or activating CASP3-mediated cleavage of CGAS or IRF3. **b**, STING1 triggers necroptosis by cooperating with downstream type I IFN-mediated MLKL expression and TNF-induced phosphorylation of RIPK1 and RIPK3. APAF1 apoptotic peptidase activating factor 1, BAK1 BCL2 antagonist/killer 1, BAX BCL2-associated X, apoptosis regulator, BCL2L1 BCL2-like 1, CASP caspase, cGAMP cyclic GMP-AMP, CGAS cyclic GMP-AMP synthase, CYCS cytochrome C, ER endoplasmic reticulum, FADD Fas associated via death domain, IFN interferon, IMM inner mitochondrial membrane, IRF3 interferon regulatory factor 3, MLKL mixed-lineage kinase domain-like pseudokinase, MOMP mitochondrial outer membrane permeability, NFKB1 nuclear factor kappa B subunit 1, RIPK1 receptor interacting serine/threonine kinase 1, RIPK3 receptor interacting serine/threonine kinase 3, STING1 stimulator of interferon response cGAMP interactor 1, TBK1 TANK binding kinase 1, TNF tumor necrosis factor

The activation of STING1 during apoptosis was first reported in 2014 by two groups. They found that mitochondrial apoptosis mediated by BCL2-associated X, apoptosis regulator (BAX), and BCL2 antagonist/killer 1 (BAK1) leads to mtDNA release and subsequently the activation of the CGAS-STING1 pathway in CASP9-deficient cells, which contributes to the inflammatory response^109,110^. Further studies confirm that BAX/BAK1-mediated MOMP is required for mtDNA-dependent STING1 activation^111,112^, while active CASP3 can cleave CGAS or IRF3 to prevent excessive IFN production^113^. Consistently, the depletion of transcription factor A, mitochondrial (TFAM, a mtDNA binding protein essential for genome maintenance) also induces mtDNA-mediated inflammation by activating STING1 in renal tubule cells^114^ (Fig. 3). Moreover, STING1 facilitates apoptotic DNA-induced pro-inflammatory gene expression in hematopoietic cells or apoptosis-derived membrane vesicle (AdMV)-induced type I IFN expression in THP-1 cells^115,116^, respectively. In addition, apoptotic tumor cells activate the STING1 pathway in tumor-associated macrophages (TAMs), thereby enhancing antitumor immune responses^117^. These studies from different groups suggest that apoptotic cells can activate STING1 to produce inflammation or an immune response.

The activation of STING1 also determines the sensitivity to apoptosis in a context-dependent manner. For example, STING1 agonists can trigger apoptosis in malignant B cells^118^, neuroblastoma cells^119^, and cancerous T cells^120^, but not in MEFs, bone marrow-derived DCs, and bone marrow-derived macrophages (BMDMs)^120^. In a sepsis mouse model, elevated mtDNA induces STING1-dependent apoptosis in intestinal epithelial cells^121^ (Fig. 3). Interestingly, both STING1 deficiency and activation promote the polarization of TAMs into a pro-inflammatory subtype, thereby inducing apoptosis in gastric cancer cells through the IL6R-JAK-IL24 pathway^122^. These data also suggest a role of STING1 signaling in mediating cell–cell communication in the tumor microenvironment.

The mechanism of STING1-mediated apoptosis is mainly related to ER stress based on several findings. First, STING1-dependent apoptosis occurs simultaneously with increases in spliced X-box binding protein 1 (XBP1, a marker of ER stress) in T cells^123^, whereas the genetic or chemical inhibition of spliced XBP1 impairs STING1-mediated apoptosis in B cells^118^. Second, the constitutive activation of STING1 by gain-of-function mutation enhances the sensitivity of T cells to apoptosis through ER-related calcium signaling^80^. Third, *Mycobacterium bovis* infection in RAW.247 cells or ethanol treatment in hepatocytes triggers ER stress and subsequent STING1-dependent apoptosis^124,125^ (Fig. 3). Structurally, STING1 activates ER stress through an evolutionarily conserved motif within CDN binding domain, which is also crucial for autophagy induction^76^. In contrast, the notch intracellular signaling domain (NICD) interacts with the CDN binding domain of STING1, thereby inhibiting STING1-dependent apoptosis in CD4^+^ T cells^126^. These results establish a link between STING1, ER stress, and apoptosis, although it is unclear whether STING1 regulates the activation of CASP12, which is an initiated caspase in ER stress-induced apoptosis.

In addition to ER signaling, STING1-mediated apoptosis may require the activation of the IRF3 pathway in some conditions. These conditions include free fatty acid-induced L-O2 cells^127^, retrovirus-infected primary human monocytes^128^, *Mycobacterium bovis*-infected RAW.247 cells^124^, LPS-induced cardiomyocytes^129^, and ethanol-treated hepatocytes^125^ (Fig. 3). Mechanistically, STING1-mediated phosphorylation of IRF3 triggers the formation of the IRF3-BAX complex, which leads to BAX activation, CYCS release, and apoptosis. These findings raise concerns about the mitochondrial function of IRF3 in innate immunity.

Taken together, the STING1-IRF3 pathway engages in both apoptosis-derived immune response and apoptosis induction, and this pathway can be regulated by apoptotic caspases, while the interplay and feedback mechanism between STING1, ER stress, and apoptosis is definitely complex.

### STING1 in necroptosis

Necroptosis is a form of regulated necrosis in which caspase is inhibited. It can be triggered by the activation of death receptors or PRRs, such as toll-like receptor 3 (TLR3), TLR4, and Z-DNA binding protein 1 (ZBP1/DAI). The core regulator of necroptosis is involved in the formation of necrosome, which is composed of three core components: receptor interacting serine/threonine kinase 1 (RIPK1), RIPK3, and mixed-lineage kinase domain-like pseudokinase (MLKL). After being activated by RIPK1 and RIPK3, phosphorylated MLKL oligomerizes and destroys the plasma membrane, leading to necroptotic cell death^130^.

The activation of STING1 is related to necroptotic stimulation. In sporadic aortic aneurysm and dissection tissue, increased stress in smooth muscle cells causes the release of mtDNA and nDNA into the cytosol, resulting in the activation of the CGAS-STING1 pathway and necroptosis^131^. A deficiency of STING1, TBK1, or IRF3 reduces, whereas the overexpression of STING1 and TBK1 increases, the phosphorylation of RIPK3 and MLKL, indicating that the STING1-IRF3-RIPK3-MLKL pathway drives necroptosis by a phosphorylation mechanism. Given that IFNB is required for RIPK3 activation and necroptosis in macrophages^132^, STING1 may also contribute to necroptosis by IFN production. Indeed, the constitutive activation of the CGAS-STING1 pathway instead of ZBP1 in macrophages elevates MLKL expression and necroptosis through IFNB^133^. In addition, BCL2 binding component 3 (BBC3/PUMA)-induced mtDNA release activates STING1-dependent necroptosis by elevating the expression of RIPK3 and MLKL in HT29 colon cancer cells and MEFs^134^ (Fig. 3). These data also indicate that MLKL is an expression of STING1-dependent ISGs, providing an additional transcription-mediated modulation mechanism of necroptosis.

However, the overexpression of MLKL alone could not restore necroptosis in IFN receptor-deficient macrophages, implying other STING1-dependent downstream products may mediate MLKL phosphorylation for necroptosis^133^. In agreement with this hypothesis, TNF production is also involved in STING1-mediated necroptosis based on four independent studies (Fig. 3). First, anti-TNF neutralizing antibody inhibits murine gammaherpesvirus-68 (MHV68)-induced necroptosis in the fibrosarcoma L929 cell line in a STING1-dependent manner^135^. Second, the activation of CGAS-STING1 in vivo and in vitro can lead to RIPK3-mediated necroptosis, which requires STING1-dependent type I IFN and TNF production^136^. Third, IL-22 induces STING1-dependent type I IFN and TNF expression in mouse small intestine organoids, driving necrosis^137^. Fourth, mtDNA-mediated STING1 signaling triggers necroptosis through synergistic IFN and TNF signaling in primary intestinal epithelial cells^138^. In sum, these data indicate that STING1 signaling triggers necroptosis through at least two mechanisms, inducing MLKL expression or MLKL phosphorylation.

### STING1 in pyroptosis

Pyroptosis is a caspase-dependent RCD driven by pore formation protein gasdermin D (GSDMD) or gasdermin E (GSDME/DFNA5). During inflammasome activation or other stresses, GSDMD can be cleaved by CASP1, CASP11 (also known as CASP4 or CASP5 in humans) or CASP8 to produce N-terminal fragment of GSDMD (GSDMD-N)^139^. In contrast, the production of GSDME-N is mediated by CASP3^140^. After oligomerization, GSDMD-N or GSDME-N forms pores in the plasma membrane, leading to pyroptotic cell death. Inflammasomes are divided into two categories: canonical and noncanonical. Canonical inflammasome complexes are assembled in response to signaling from cytosolic PRRs, such as NLR family pyrin domain containing 3 (NLRP3) and absent in melanoma 2 (AIM2). These PRRs sense PAMPs or DAMPs to recruit the adapter protein PYD and CARD domain containing (PYCARD/ASC), leading to CASP1 activation and subsequent pyroptosis^141^. Noncanonical inflammasome is mainly triggered by cytoplasmic LPS-induced CASP11 or CASP4/5 activation, resulting in the production of GSDMD-N and pyroptosis^142^. As a feedforward control, the GSDMD pores allow potassium efflux, promoting the activation of NLRP3 inflammasome, which then causes the release of IL1 family cytokines or DAMPs.

**Fig. 4:**
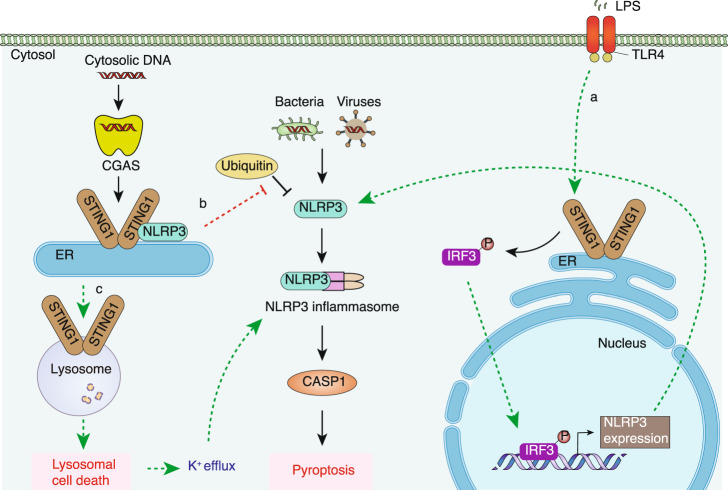
STING1 in pyroptosis. STING1 activates NLRP3 and subsequent pyroptosis via three pathways: **a** LPS directly triggers STING1-IRF3 activation via TLR4 and activated IRF3 increases the expression of NLRP3; **b** STING1 interacts with NLRP3 and prevents ubiquitin-mediated degradation of NLRP3; **c** STING1 induces lysosomal cell death, causing potassium efflux to activate an NLRP3 inflammasome. CASP caspase, CGAS cyclic GMP-AMP synthase, ER endoplasmic reticulum, IRF3 interferon regulatory factor 3, LPS lipopolysaccharide, NLRP3 NLR family pyrin domain containing 3, STING1 stimulator of interferon response cGAMP interactor 1, TLR4 toll-like receptor 4

STING1 promotes cGAMP-induced NLRP3 inflammasome activation in macrophages^143^, cardiomyocytes, and mice^129^ following LPS challenge. In addition, *Mycobacterium abscessus* infection in murine macrophages triggers mitochondrial oxidative stress and leads to the activation of the CGAS-STING1 pathway and NLRP3 inflammasome activation^144^, while the inhibition of STING1 suppresses NLRP3 activation in mtDNA-stimulated BMDMs from aged mice^145^. In terms of mechanisms, three pathways may contribute to STING1-dependent inflammasome activation and pyroptosis (Fig. 4). First, the transcriptional upregulation of NLRP3 requires the production of STING1-mediated IRF3 in a TLR4-dependent manner^129^. Second, STING1 can directly bind and promote NLRP3 localization in the ER, thereby inhibiting NLRP3 ubiquitin degradation^146^. Third, in response to cytosolic DNA in human myeloid cells, the CGAS-STING1 pathway instead of AIM2 activates potassium efflux-mediated NLRP3 inflammasome via lysosomal STING1-mediated lysosomal cell death^147^. Consistent with this, G10 (an agonist of STING1) induces potassium efflux and NLRP3 activation in porcine cells^148^. Conversely, the inhibition of NLRP3 inflammasomes increases STING1-dependent IFN production^148^, while the activation of NLRP3 or AIM2 in murine macrophages leads to the inactivation of the STING1 pathway^149,150^, suggesting a yet unknown mechanism to balance the activation of STING1 and inflammasomes in response to infection.

### STING1 in ferroptosis

Ferroptosis is a type of regulated necrosis^151^, which is characterized by iron-dependent lipid peroxidation and was first described as a mutant RAS-dependent cancer cell death^152^. In addition to cancer^153^, ferroptotic injury is related to various tissue injuries and infections^154–156^. Ferroptosis can be induced by destroying certain antioxidant systems, especially the SLC7A11-glutathione (GSH)-GPX4 axis^152^. Ferroptosis may be a type of ADCD because several key anti-ferroptosis regulators^157^, such as ferritin^65^, aryl hydrocarbon receptor nuclear translocator-like (ARNTL)^158^, GPX4^159^, solute carrier family 40 member 1 (SLC40A1, best known as ferroportin)^68^, and lipid droplets^160^, can be eliminated by autophagic pathways.

**Fig. 5:**
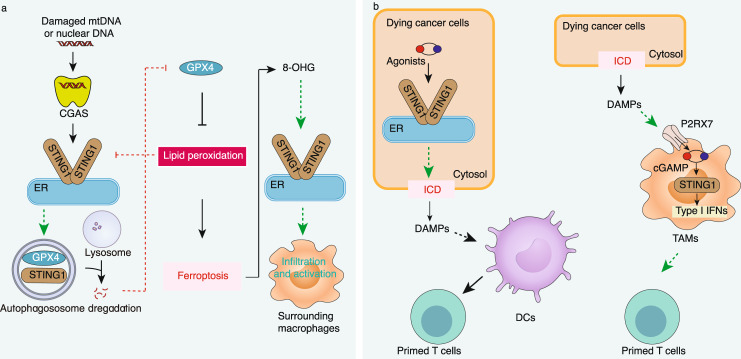
STING1 in ferroptosis and ICD. **a** mtDNA and nDNA activate STING1-dependent autophagy, leading to GPX4 degradation and subsequent lipid peroxidation-mediated ferroptosis, whereas lipid peroxidation negatively regulates STING1 through carbonylation. Ferroptotic damage also activates STING1 in surrounding macrophages by releasing oxidative DNA damage products, such as 8-OHG. **b** STING1 engages in ICD in two ways: inducing DC-dependent T-cell priming or TAM-mediated T-cell priming. 8-OHG 8-hydroxy-2′-deoxyguanosine, cGAMP cyclic GMP-AMP, CGAS cyclic GMP-AMP synthase, DAMPs damage-associated molecular patterns, DCs dendritic cells, ER endoplasmic reticulum, GPX4 glutathione peroxidase 4, ICD immunogenic cell death, P2RX7 purinergic receptor P2X 7, STING1 stimulator of interferon response cGAMP interactor 1, TAMs tumor-associated macrophages

Recently, STING1 has been connected to GPX4-mediated lipid peroxidation in the context of ferroptosis^161,162^. GPX4 depletion increases lipid peroxidation in HSV-1 infected mice, thereby limiting STING1-mediated antiviral immune responses^161^. One reason is that the lipid product 4-hydroxynonenal (4-HNE) inhibits STING1 activation by the carbonylation of STING1 in mouse primary peritoneal macrophages, suggesting that GPX4 may act as a promoter of STING1-mediated immune response during virus infection. However, the conditional depletion of GPX4 in myeloid cells increases death caused by bacterial infection in mice, suggesting a different role of GPX4 in bacterial innate immunity^163^. Moreover, mitochondrial or genomic DNA stress activates STING1-dependent autophagy in pancreatic cancer cells, which may cause lipid peroxidation-mediated ferroptosis^70,71^. Although the mechanism remains obscure, STING1-dependent autophagy may degrade GPX4, promoting ferroptosis^71^ (Fig. 5). Accordingly, a robust STING1 activation produces excessive lipid peroxidation during ischemia/reperfusion injury, which is associated with cell death in BMDMs^164^. Thus, the STING1-related ferroptosis pathway is a potential therapeutic target of cancer and tissue damage.

STING1 plays a dual role in tumor immunity. An acute activation of STING1 is beneficial for antitumor therapy, and chronic activation of STING1 may mediate inflammation that supports tumor growth. Consistent with this idea, ferroptotic damage promotes 8-hydroxy-2′-deoxyguanosine (8-OHG, an oxidized nucleobase product of oxidative DNA damage) release, thereby activating STING1-dependent macrophage polarization^162^. By maintaining inflammation-related tumorigenesis, this chronic activation of STING1 triggered by ferroptotic DNA damage ultimately promotes KRAS-driven pancreatic ductal adenocarcinoma in mice (Fig. 5), highlighting the dark side of ferroptosis in tumor immunity^165^. Further research is needed to clarify the role of STING1 in regulating ferroptosis in different immune cells within the tumor microenvironment.

### STING1 in mitotic death

Mitotic death is a specific variant of RCD driven by mitotic catastrophe, which suppresses the proliferation of cells undergoing aberrant mitosis^60^. It usually ends with the formation of large cells with multiple micronuclei and decondensed chromatin^166^. Ruptured micronuclei can be recognized by CGAS during genome instability to activate an innate immune response^167,168^. To avoid hyperactivation, CGAS is normally inhibited by nucleosomes^169–173^.

During mitotic arrest, CGAS triggers the activation of STING1 and IRF3 in response to the increase of micronuclei, which leads to mitotic death/apoptosis by blocking BCL2-like 1, (BCL2L1/BCL-XL)-dependent MOMP inhibition (Fig. 3)^174,175^. As discussed previously, increased telomeric DNA damage or *Burkholderia pseudomallei* infection also causes STING1-dependent mitotic death, which is related to the excessive activation of autophagy^74,75^. These data implicate STING1-mediated mitotic death as an early host defense against tumorigenesis or infection.

### STING1 in immunogenic cell death

ICD is implicated in generating antitumor adaptive immunity^95^. During ICD, dying cells release or expose DAMPs, activate DC-mediated antigen presentation, and ultimately lead to cytotoxic T-cell responses. ICD is associated with multiple cell death modalities (e.g., apoptosis, pyroptosis, necroptosis, and ferroptosis) and can be triggered via a set of therapeutic agents and interventions.

ICD is also induced in some cancer cells by activating the CGAS-STING1 pathway. The effective activation of STING1 by various agonists stimulates ICD-mediated antitumor immunity in colon carcinoma cells^176^, neuroblastoma cells^119^, and melanoma cells^177^ by producing highly immunogenic cancer cell debris or type I IFNs (Fig. 5). Moreover, DNA released from apoptotic MC38 tumor cells can stimulate a STING1-dependent type I IFN response in TAMs (but not DCs) through purinergic receptor P2X 7 (P2RX7) channels, thereby further enhancing antitumor CD8^+^ T-cell response^117^ (Fig. 5). Given that CGAS is constitutively active in most tumor cells and results in the production of cGAMP, which can be internalized by bystander cells through gap junctions^13^, transporter solute carrier family 19 member 1 (SLC19A1)^14^, or volume-regulated anion channels^15,178^, targeting the STING1 pathway has the potential to overcome the immunosuppressive tumor microenvironment.

## Conclusions and perspectives

**Fig. 6:**
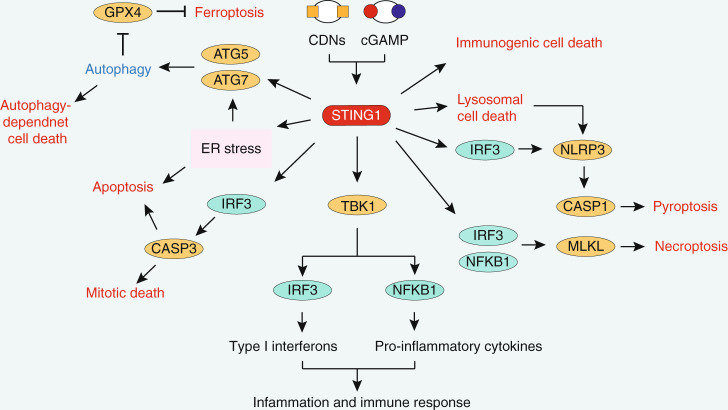
The network of STING1 in inflammation, immune response, autophagy, and cell death. STING1 can be activated by bacteria-produced CDNs or CGAS-produced cGAMP. The activation of STING1 not only promotes inflammation and immune responses through TBK1-mediated activation of transcription factor, but also contributes to autophagy and various cell death modalities, such as apoptosis, necroptosis, pyroptosis, ferroptosis, mitotic death, immunogenic cell death, and autophagy-dependent cell death. GPX4 glutathione peroxidase 4, CDNs cytosolic cyclic dinucleotides, cGAMP cyclic GMP-AMP, ATG autophagy-related, STING1 stimulator of interferon response cGAMP interactor 1, ER endoplasmic reticulum, IRF3 interferon regulatory factor 3, NLRP3 NLR family pyrin domain containing 3, TBK1 TANK binding kinase 1, CASP caspase, NFKB1 nuclear factor kappa B subunit 1, MLKL mixed-lineage kinase domain-like pseudokinase

In the past 5 years, by combining genetic technology and pharmacological approaches (Table 1), our understanding of the regulation and function of STING1 has rapidly improved (Fig. 6). Accordingly, there is growing interest in the development of natural and synthetic CDN analogs as well as non-CDN small molecule STING1 agonists as clinical drugs for cancer treatment and antiviral therapy (Table 1). However, because tumor-specific T cells can initiate immunosuppressive pathways including CD274 molecule (best known as PD-L1), thereby preventing tumor clearance^179^, activating the STING1 pathway alone (e.g., using cyclic diadenyl monophosphate) is not sufficient to kill tumors^180,181^. Therefore, the combined use of STING1 agonists (e.g., using ADU-S100) and immune checkpoint inhibitors may be the best strategy for tumor treatment^182,183^. In addition, STING1 activation can impair immunotherapy because it is a mediator of IFN-induced cell death of B cells^184^ or T cells^185^. Consequently, STING1 inhibitors (Table 1) can alleviate the side effects of STING1 overactivation.

**Table 1:** STING1 activators and inhibitors

Drugs	Targets	Effect	Model/Disease/Cancer Type	Clinical Trial Phase	Clinical Trial ID/Publication Number	References
**Natural CDN agonists**						
c-di-GMP	hSTING1; mSting1	Antitumor activity	4T1 and B16 mouse models			^186,187^
2′,3′-cGAMP	hSTING1; mSting1	Antitumor activity	CT26, 4T1, and B16F10 mouse models			^188^
**Synthetic CDN agonists**						
ML-RR-S2-CDA	hSTING1 mSting1	Antitumor activity	B16F10, 4T1, and CT26 mouse models			^189^
ML-RR-S2-CDG	hSTING1 mSting1	Antitumor activity	B16F10 mouse models			^189^
3′,3′-cAIMP	hSTING1 mSting1	Antiviral activity	HSV2 infection			^190^
**Non-CDN agonists**						
DMXAA	mSTING1	Antitumor activity	Various mouse models	Failed in phase III clinical trial		^191^
FAA	mSTING1	Antitumor activity	Various mouse models	Failed in phase I clinical trial		^192^
CMA	mSTING1	Antiviral activity	Murine models			^193^
α-Mangostin	hSTING1; mSting1	Antitumor and antiviral activity				^194^
ABZI	hSTING1; mSting1	Antitumor activity	CT26 mouse models			^195^
Benzothiophenes	hSTING1; mSting1	Antitumor activity	MC38 mouse models		WO2019027858	
MSA-2	hSTING1; mSting1	Antitumor activity	MC38 mouse models			^196^
SR-717	hSTING1; mSting1	Antitumor activity	B16F10 mouse models			^197^
**STING1 agonists currently in clinical trials**						
ADU-S100	Synthetic CDN analog	Antitumor activity	B16 mouse models	Failed in phase II clinical trial		^198^
ADU-CL-20	Synthetic CDN analog	Antitumor activity	Metastatic/recurrent HNSCC	Phase II	NCT03937141	
MK-1454	Synthetic CDN analog	Antitumor activity	Advanced solid tumors or lymphomas	Phase I	NCT03010176	
MK-2118	Synthetic CDN analog	Antitumor activity	Advanced solid tumors or lymphomas	Phase I	NCT03249792	
BMS-986301	Undisclosed	Antitumor activity	Advanced solid cancers	Phase I	NCT03956680	
GSK3745417	diABZI-like	Antitumor activity	Advanced solid tumors	Phase I	NCT03843359	
SB-11285	CDN analog	Antitumor activity	Advanced solid tumors	Phase I	NCT04096638	
IMSA-101	cGAMP analog	Antitumor activity	Advanced solid tumors	Phase I/II	NCT04020185	
E7766	Synthetic CDN analog	Antitumor activity	Advanced solid tumors; lymphomas	Phase I	NCT04144140	
**STING1 inhibitors**						
H-151	Blocks palmitoylation of STING1					^199^
C-178	Blocks palmitoylation of STING1					^199^
C-176	Blocks palmitoylation of STING1					^199^
C18	Blocks cGAMP-induced IFNB production					^200^
Astin-C	Blocks recruitment of IRF3 to STING1					^201^

In addition to the traditional function of STING1 in mediating inflammation and immune response, emerging evidence has revealed that STING1 is a key regulator of autophagy and cell death after its post-translational modification. For example, palmitoylation at C88/91 residues of STING1 is not only essential for maintaining active STING1 on Golgi^23^, but also for STING1-mediated T cell death^185^. K63 or K48-linked STING1 ubiquitination may be involved in SQSTM1-related autophagy^76^. Generally, HeLa, MEF, HEK-293T, THP-1, and BMDM are widely used cell lines, while STING1^−/−^ mice or xenograft models are widely used animal models to study the function of STING1 in autophagy and cell death. The complex cellular and immune function of STING1 is achieved through its location, modification, and protein–protein interaction. Overall, the activation of STING1 promotes autophagy and mediates many types of cell death, thus highlighting the important role of STING1 in integrating inflammation and immune response under various stresses. Accordingly, the development of STING1 agonists and inhibitors has become a frontier for the treatment of diseases by stimulating or suppressing the immune response. The following questions are worthy of our continued pursuit: Does STING1-dependent control of autophagic signaling contribute to the maintenance of a stress threshold? If STING1 is an autophagy receptor, what is its core substrate? Why does STING1 share a common upstream activation signal during DNA damage but can then lead to different cell death pathways? In addition to the ER, what is the function of STING1 in other subcellular organelles? How do autophagy and cell death pathways combine to control the immune response? How can we evaluate the difference between CGAS-dependent and independent STING1 pathway activation in host defense? Are there any specific biomarkers that can be used to monitor STING1-dependent autophagy and cell death? What are the activities and side effects of different STING1 drugs? How can we develop a STING1-dependent combination drug strategy for tumor treatment?
